# The (neuro)cognitive mechanisms behind attention bias modification in anxiety: proposals based on theoretical accounts of attentional bias

**DOI:** 10.3389/fnhum.2013.00119

**Published:** 2013-04-04

**Authors:** Alexandre Heeren, Rudi De Raedt, Ernst H. W. Koster, Pierre Philippot

**Affiliations:** ^1^Laboratory for Experimental Psychopathology, Psychological Sciences Research Institute, Université Catholique de LouvainLouvain-la-Neuve, Belgium; ^2^Psychopathology and Affective Neuroscience Lab, Department of Experimental Clinical and Health Psychology, Ghent UniversityGhent, Belgium

**Keywords:** attentional bias, cognitive bias modification, experimental psychopathology, neuromodulation, DLPFC

## Abstract

Recently, researchers have investigated the causal nature of attentional bias for threat (AB) in the maintenance of anxiety disorders by experimentally manipulating it. They found that training anxious individuals to attend to non-threat stimuli reduces AB, which, in turn, reduces anxiety. This effect supports the hypothesis that AB can causally impact the maintenance of anxiety. At a fundamental level, however, uncertainty still abounds regarding the nature of the processes that mediate this effect. In the present paper, we propose that two contrasting approaches may be derived from theoretical accounts of AB. According to a first class of models, called the “valence-specific bias” models, modifying AB requires the modification of valence-specific attentional selectivity. According to a second class of models, called the “attention control models,” modifying AB requires the modification of attention control, driven by the recruitment of the dorsolateral prefrontal cortex. We formulate a series of specific predictions, to provide suggestions to trial these two approaches one against the other. This knowledge is critical for understanding the mechanisms of AB in anxiety disorders, which bares important clinical implications.

## Introduction

**Figure 1:**
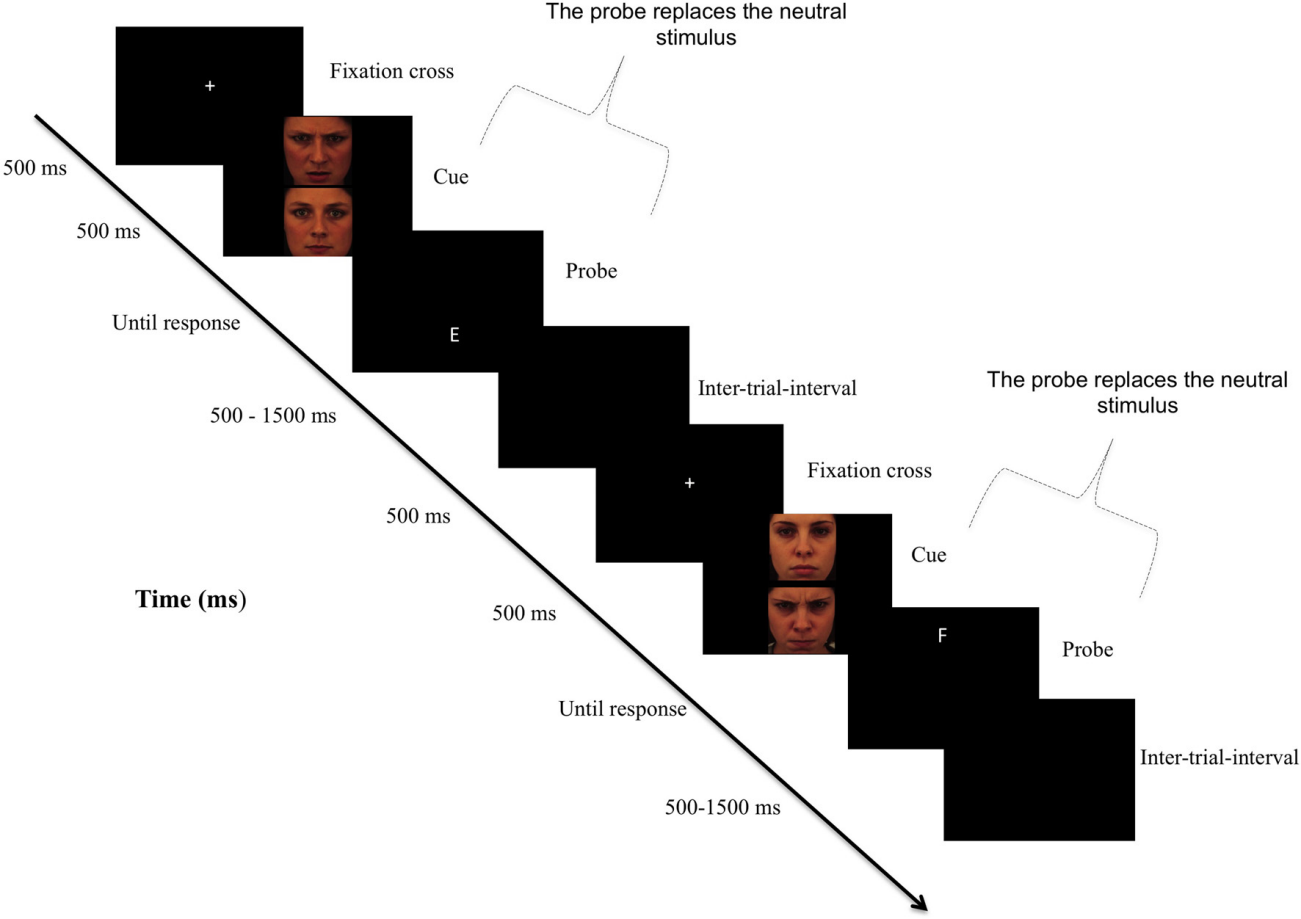
**Sequence of the attention bias modification procedure.** Note: In the original version of the dot-probe paradigm, participants viewed two stimuli (i.e., a threatening and a neutral) presented in two areas of a computer screen for approximately 500 ms. Immediately after the pictures disappeared, a probe replaced one of the stimuli. Participants responded to the probe as quickly as possible. In attention training, researchers typically modify the original task such so that the probe nearly always (i.e., 95% of the trials) replaces the neutral stimulus, thereby redirecting subjects' attention to non-threat cues. In the control condition, there was no contingency between cues and probes.

The ability to rapidly orient attention toward threat in the environment is crucial for survival. A wealth of research has demonstrated that this phenomenon is exacerbated in anxiety disorders (Bar-Haim et al., 2007), where anxious individuals demonstrate faster engagement with and/or impaired disengagement from threatening stimuli (Cisler and Koster, 2010). Recently, researchers have investigated the causal nature of these biases in the *maintenance* of anxiety disorders, by directly manipulating attentional biases (AB) for threat. They found that training anxious individuals to attend to non-threat cues (see Figure 1) reduces AB which, in turn, reduces anxiety (Hakamata et al., 2010; Beard et al., 2012). This effect supports the hypothesis that AB can causally influence the maintenance of anxiety. At a fundamental level, however, uncertainty abounds regarding the nature of this process. Clarifying the mechanisms involved is important as attention bias modification (ABM) has only limited effectiveness in changing the processing of threat (Beard et al., 2012), which is crucial for studies using ABM for causal predictions as well as therapeutic applications.

The main goal of the present paper is to relate ABM to the neurocognitive mechanisms proposed by theoretical accounts of AB^1^. We provide a brief review of theoretical and empirical insights on these mechanisms. Then, we formulate suggestions about how future research may reduce uncertainty regarding these mechanisms.

## Theoretical accounts of AB: state of the art

Different models have been advanced to account for the mechanism underlying the role of AB in the maintenance of anxiety. We discuss here the main proposals by these models, which we have organized into two broad approaches.

### Valence-specific models

According to a first approach, AB is the result of valence-specific cognitive operations at the level of appraisal and attention. Different models propose such a rationale (e.g., Beck and Clark, 1997; Mathews and Mackintosh, 1998; Mogg and Bradley, 2002). For instance, according to the cognitive-motivational model of anxiety (Mogg and Bradley, 1998, 2002), allocation of attention is a function of a valence evaluation system (VES), which is in charge of an initial appraisal of the stimulus threat value. Similarly, according to Mathews and Mackintosh's model (1998), AB is the result of a threat evaluation system that strengthens the activation of threat-related attributes. Finally, according to Beck and Clark (1997), AB results from an initial threat appraisal, which favors the processing of threat-related material, and in turn leads to the activation of cognitive, physiological, and behavioral anxious responses.

Considered together, these models share the feature that anxious individuals are characterized by a heightened threat evaluation, even when the true threat value of the stimulus is mild or ambiguous. This appraisal is the driving factor behind subsequent AB. Research has found indirect support for the main claim of these models, showing that high anxious individuals are indeed more likely to allocate attention to mildly threatening information (Wilson and MacLeod, 2003). However, data on initial threat appraisal, potentially providing direct support for this claim, is inconsistent. For instance, it has been reported that socially anxious individuals do not show biased evaluation of threatening facial expressions (e.g., Melfsen and Florin, 2002; Schofield et al., 2007) or of emotional facial expressions in general (Philippot and Douilliez, 2005). However, recent findings did show that socially anxious participants process early configural information^2^ differently than did the non-anxious participants (Langner et al., 2009).

The idea of a valence-specific bias also fits with some of the neural data showing enhanced amygdala activity [and functionally-related structures, for a review see Hofmann et al. (2012)] involved in AB (Davis and Whalen, 2001). More centrally, a wealth of data has demonstrated that the amygdala is involved in early threat detection mechanisms (Öhman, 2005) and that anxious individuals have increased amygdala activation in response to threat (Stein et al., 2002; Phan et al., 2006). Alongside research focusing explicitly on AB, event-related brain potentials (ERP) studies, in which early components (e.g., P1) are usually linked to configural mechanisms, while later components (e.g., P2, P3) are linked to more strategic and executive mechanisms, also revealed early signatures of visual processing of threat, i.e., change in early posterior negativity, N170, and in P1 (Vuillemier and Pourtois, 2007; Rossignol et al., 2012).

### Attentional control models

According to a second approach, AB may be considered as the result of impaired attention control (AC), i.e., the ability to voluntarily regulate the allocation of attentional resources. This notion is based on findings that AC modulates AB. For instance, Derryberry and Reed (2002) have found that high-trait anxious individuals reporting poor AC exhibited stronger AB in a spatial cueing task. In contrast, those who reported good AC did not exhibit such effect. Similar findings were found using rapid serial visual presentation task (Peers and Lawrence, 2009).

Different neurocognitive models have provided an explanation for these findings (Bishop, 2007; Eysenck and Derakshan, 2011). First, according to Attention Control Theory (Eysenck and Derakshan, 2011), impairments in the efficiency of the central executive, particularly the inhibitory function of the central executive (Eysenck et al., 2007) may maintain AB and anxiety. Evidence for this position comes from studies showing that clinically anxious individuals exhibit worse performance in tasks requiring such control (e.g., antisaccade task), even in the absence of threatening material (Derakshan et al., 2009; Wieser et al., 2009). For instance, Wieser et al. (2009) reported that socially anxious individuals exhibit difficulties in inhibiting the reflexive orienting to neutral as well as to emotional stimuli.

Second, according to Bishop's model (Bishop et al., 2004; Bishop, 2008, 2009), AB can be seen as a failure to recruit AC, and this failure is associated with decreased activation of the prefrontal cortex, particularly of its dorsolateral part (DLPFC) in order to down-regulate amygdala activation during the presentation of threat (with increased amygdala activity as a proxy of output from the VES). Accordingly, brain imaging studies show that anxious individuals demonstrate reduced activation of the DLPFC during such an inhibitory task (Bishop, 2009). However, as these studies did not include any connectivity analysis, conclusions can only be drawn based on prefrontal activation patterns. Future studies should further explore whether the DLPFC—amygdala connectivity is associated with the failure to recruit AC.

## Relating ABM to the theoretical accounts of AB

Remarkably, there is extensive theorizing on the mechanisms associated with AB but ABM research is rarely related to these theories. In relation to key models of anxiety and attention depicted above, two core hypotheses can be generated to account for the effect of ABM on anxiety.

According to the first class of models, the *valence-specific models* (VSM) the maintenance of AB is the result of the activation of a biased VES. These models propose that, regardless of attentional resources, individuals would allocate attention to stimuli that are initially appraised as threatening. According to such models, if the VES remains unchanged individuals should initially orient to threat.

According to the alternative models, hereafter called *attention controlmodels* (ACM), AB involves impaired recruitment of AC. These latter models propose that anxious individuals are characterized by an impaired recruitment of AC in cognitively demanding tasks, which is most pronounced in the presence of external threat or anxious thoughts. In relation to such models, the reduction of AB involves an improvement in the AC system without necessarily changing the valence-specific bias.

## Theoretical formulations and predictions

**Figure 2:**
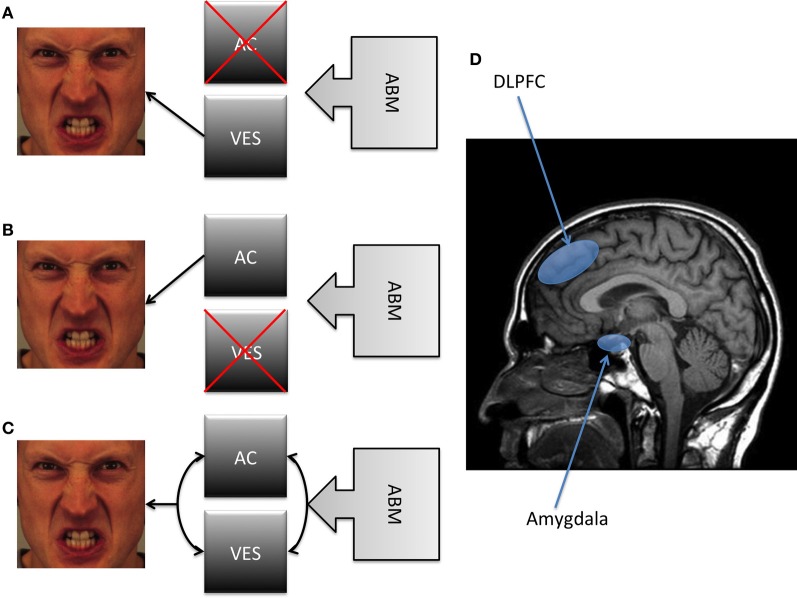
**A schematic summary of the different models depicted in the present article.** Note: **(A)** A first position proposes a strong causal antecedent role for the VES, claiming that the reduction of AB necessarily involves a modification of the valence-specific bias without involving an improvement in AC. **(B)** In contrast, a second position suggests that ABM involves an improvement in AC without any modification of the VES. **(C)** A third position suggests that both VES and AC are causally involved in the reduction of AB. **(D)** It has been suggested that the DLPFC may be considered as a proxy of AC, whereas the amygdala as a proxy of the VES. Abbreviations: AB, Attentional Bias; AC, Attention Control; DLPFC, Dorsolateral Prefrontal Cortex; VES, Valence Evaluation System.

We argue that these two types of processes can be contrasted to consider the mechanisms underlying the therapeutic benefits of ABM. According to VSM, modifying AB should not influence early AB as this is under control of the VES. According to ACM, modifying AB requires the modification of AC. Although both approaches share the view that AB is a factor causally involved in the maintenance of anxiety, the nature of the process leading to this involvement is still unclear. A future challenge would be to specify how these two types of processes are involved in the maintenance of AB. At a basic level, they differ on at least two dimensions (see Figure 2). One dimension is the requirement of AC. VSM holds that the implication of AC does not determine the reduction of AB, whereas ACM predicts that reducing AB requires such a reduction. However, to our knowledge, no previous study has directly assessed the impact of directly manipulating AC on the effect of AB.

Another critical dimension is the function of the VES. VSM hold a strong causal antecedent position concerning the VES, suggesting that the reduction of AB necessarily involves a modification of the valence-specific bias without necessarily involving an improvement in AC. In contrast, the ACM suggests that ABM involves an improvement in general AC without involving a modification of the VES.

Finally, even if the two theoretical accounts discussed above are presented as opposing each other, it is important to mention that the mechanisms suggested by VSM and ACM interact during ABM. Research has found that “emotional” areas may inform “cognitive” areas about the value of directing attention to a given stimulus (e.g., Pessoa and Engelmann, 2010). Conversely, AC may change the responsivity of “emotional” areas, such as the amygdala (Pessoa, 2008, 2010). Moreover, the activation of the amygdala toward threat depends on the availability of the attention resources (Pessoa, 2005), highlighting again the fuzzy boundaries between the VES and AC.

As a consequence, a first central issue is the exploration of the involvement of AC in ABM. According to the ACM, reducing AB should require such a control process whereas this is not necessary according to VSM. Previous researchers have hypothesized that ABM bolsters AC in ways that may foster the ability to down regulate anxiety (Klumpp and Amir, 2010). Moreover, it has also been reported that change in AB via ABM depends on the initial level of AC (Paulewicz et al., 2012). Further, supporting the hypothesis that frontal regions (as a proxy of AC) are involved in ABM, it has been shown that inducing AB for threat is related to altered activation of the DLPFC to emotional stimuli rather than to change in subcortical regions (Browning et al., 2010). Despite this preliminary suggestion, the general AC hypothesis needs to be tested by directly manipulating the involvement of such a control mechanism *during* ABM. To test this hypothesis, future studies may cross an ABM procedure with a manipulation of the cognitive load using a dual-task impairing the central executive resources (e.g., memorizing 1 number/low load vs. memorizing 6 numbers/high load). If the VSM hypothesis is correct, then AB should decrease in both ABM conditions regardless of the availability of attentional resources. In contrast, if the ACM hypothesis is correct, then AB should be reduced in the low cognitive load condition compared to the high cognitive load condition.

Second, all the theoretical perspectives predict differences with regard to the benefits of ABM. VSM predicts that leaving the VES unaltered, early AB will still be observed, whereas ACM predicts that initial attentional interference of threat can be reduced by improving AC. Using ERP experiments that can shed light on the time-course processing of AB, Eldar and Bar-Haim (2010) found that ABM reduced P2 and P3 amplitudes and increased N2 amplitude in response to the onset of threatening stimuli during a dot-probe task. They interpreted these data as implying that ABM involves late executive mechanisms rather than early ones. In accordance with this hypothesis, Koster et al. (2010) found that ABM influences late (1500 ms) rather than early (30 or 100 ms) stages of threat processing. Again, despite these preliminary data, this hypothesis is in need of experimental manipulations directly investigating the implication of the VES in the maintenance of AB.

## Future research avenues

Through carefully considering how models of AB can inform training approaches, two innovative lines of research appear promising to advance ABM.

### Combined modification of threat appraisal and attention

One interesting way to disentangle different models of attention involvement in anxiety, as well as to potentially strengthen the effects of ABM, could be to combine different training procedures. According to VSM, reducing the sensitivity of the VES to threatening stimuli should reduce AB, whereas ACM does not make this prediction. To examine this hypothesis, future experiments could simultaneously manipulate VES and AC. Previous research has shown that both AC (e.g., Klingberg, 2010) and VES (e.g., Clerkin and Teachman, 2010) are malleable. Regarding AC, for instance, it has been shown that such control can be increased over time using a repetitive training with cognitive training tasks (Olesen et al., 2004). Based on previous studies, the VES may be manipulated using a procedure based either on a classical evaluative conditioning paradigm, in which a threatening picture is paired with a more positive picture to reduce the evaluative meaning of the threat picture (Clerkin and Teachman, 2010) or on extinction paradigms in which a conditioned stimulus loses it meaning in a novel context (Engelmann and Hein, 2013). It has been found that such a procedure may alter functional connectivity of the early visual processing regions (V1–V4) of threat (Damaraju et al., 2009).

Nevertheless, as such, these expected observations cannot sustain the conclusion that a change in AB can be unambiguously attributed to a change in AC or VES processing resulting from the training. As argued by MacLeod et al. (2009), this conclusion requires that studies confirm predicted changes on a task that reliably measures the mediating cognitive process. Moreover, MacLeod et al. (2009) also argue that the magnitude in change in the mediating process should predict the magnitude of improvement on the outcome measures, which is AB in this case. To address these requirements, changes in VES could be assessed using an Affective Priming Task that allows for the indirect assessment of the valence of stimuli by comparing its influence as a prime on congruently or incongruently-valenced subsequent stimuli. Changes in AC may be assessed using a general task assessing the ability to inhibit a prepotent response (e.g., antisaccade task).

If the VEM hypothesis is correct, then reducing the impairment in VES should lead to a decrease in AB. In contrast, if the ACM hypothesis is right, then improving AC should reduce AB. Finally, if both VES and AC are involved in AB, then the conditions improving either VES or AC should reduce AB, as opposed to control conditions that would present the same stimuli but that do not affect VES nor AC.

### Combining attentional bias modification and neuromodulation

According to the ACM, reducing AB should require DLPFC activation, whereas according to VEM it should not. Regarding the empirical literature, it has been shown that inducing AB for threat is related to altered activation of the DLPFC (Browning et al., 2010). Moreover, a single session of High Frequency-repetitive Transcranial Magnetic Stimulation (HF-rTMS) on this region impacts the magnitude of AB (e.g., Leyman et al., 2009; De Raedt et al., 2010). Low-frequency rTMS (LF−rTMS) of ≤ 1 Hz temporally suppresses local neural activities, while high-frequency rTMS (HF−rTMS) of ≥ 5 Hz temporally activates local neural activities (e.g., Pascual-Leone et al., 1998). As a consequence, in order to more directly examine the hypothesis of a need of DLPFC recruitment for reducing AB, future experiments in the field may use LF-rTMS vs. HF-rTMS to temporarily decrease vs. increase DLPFC (BA 9/46) activation before ABM. If the VSM hypothesis is correct, then AB should be reduced for both ABM conditions, regardless of the activation of the DLPFC. In contrast, if the ACM hypothesis is correct, then AB should only decrease for the ABM with DLPFC activation. More recently, a series of papers using transcranial direct current stimulation (tDCS) as a neuromodulation technique during cognitive task has been published (e.g., Penolazzi et al., 2012). This technique applies a weak (0.5–2 mA), direct electric current through electrodes positioned over one's scalp, which are able to reach the neuronal tissue and induce polarization-shifts on the resting membrane potential (Nitsche et al., 2008). Anodal stimulation generally facilitates cortical activity, whereas cathodal tDCS has opposite effects. The advantage of tDCS is that it allows to directly modulate cortical activities during a task. With HF-rTMS it is not possible to perform stimulation during the task, given that the effects of stimulation only emerge after the procedure. Again, future experiments in the field may use tDCS in order to directly modulate the cortical excitability of the DLPFC (localized via F3 in the international 10–20 EEG system) during ABM.

## Conclusions

Research has shown that training anxious individuals to attend to non-threat stimuli reduces AB, which, in turn, reduces anxiety. This effect supports the hypothesis that AB can causally influence the maintenance of anxiety. At a fundamental level, however, uncertainty still abounds regarding the nature of this process. Here, ABM seems only scarcely informed by key models of AB. We have argued that these models can provide critical insights on how to understand the effects of ABM. Moreover, ABM studies can be used to study the major proposals of these models.

### Conflict of interest statement

The authors declare that the research was conducted in the absence of any commercial or financial relationships that could be construed as a potential conflict of interest.

## Footnotes

^1^During the last decade, there has been increasing interest in the establishment of whether AB in anxiety results from preferential attention engagement with emotionally threatening stimuli, or from a selective difficulty in disengaging attention from such information. Researchers have developed a number of attention assessment tasks in an attempt to measure these processes. Although a growing body of research provided support for the hypothesis that anxious individuals have difficulty in disengaging attention from threat (for a review, see Cisler and Koster, 2010), it has been recently argued that many of the tasks used to assess theses mechanisms may not be able of indexing the attentional processes they claim to measure (for a review, see Clarke et al., 2013). Moreover, most of the theoretical accounts of AB did not make specific predictions about the role of attentional engagement and disengagement in the maintenance of AB. As the main focus of the present paper pertains to the neurocognitive mechanisms behind ABM that are based on theoretical accounts of AB, we used AB as a generic term for both attention engagement with emotionally threatening stimuli and from selective difficulty disengaging attention from such information (for recent studies discussing the potential role of attentional engagement and/or disengagement during ABM, see Heeren et al., 2011, 2012; Hirsch et al., 2011).

^2^Although socially anxious individuals performed as well as non-anxious individuals on a task assessing the ability to discriminate angry faces from neutral faces, they did not utilize the same facial expression for the task. The fine details (high spatial frequencies) around the eyes were discriminative for both groups, but only socially anxious participants additionally processed rough configural information (low spatial frequencies information from eyes, nose, and mouth regions).
